# *Robbsia betulipollinis* sp. nov., Isolated from Pollen of Birch (*Betula pendula*)

**DOI:** 10.1007/s00284-023-03344-7

**Published:** 2023-06-06

**Authors:** Haoran Shi, Binoy Ambika Manirajan, Stefan Ratering, Rita Geissler-Plaum, Sylvia Schnell

**Affiliations:** 1 Institute of Applied Microbiology, Research Center for BioSystems, Land Use, and Nutrition (IFZ), Justus-Liebig University Giessen, 35392 Giessen, Germany; 2 School of Biosciences, Mahatma Gandhi University, Kerala, India

## Abstract

One gram-negative strain designated Bb-Pol-6^ T^ was isolated from birch (*Betula pendula*) pollen at Giessen area, Germany. The analysis of 16S rRNA gene-based phylogenies indicated the next-relative genera were *Robbsia*, *Chitinasiproducens*, *Pararobbsia* and *Paraburkholderia* (96–95.6%). Further comparative genome analysis and phylogenetic tree-based methods revealed its phylogenetic position under the genus *Robbsia*. The genome of strain Bb-Pol-6^ T^ was 5.04 Mbp with 4401 predicted coding sequences and a G + C content of 65.31 mol%. Average amino acid identity, average nucleotide identity, digital DNA–DNA hybridization and percentage of conserved proteins values to *Robbsia andropogonis* DSM 9511^ T^ were 68.0, 72.5, 22.7 and 65.85%, respectively. Strain Bb-Pol-6^ T^ was rod-shaped, non-motile, facultative anaerobic and grew optimally at 28 °C and pH 6–7. Ubiquinone 8 was the major respiratory quinone and the major cellular fatty acids were C_16:0_, C_19:0 cyclo_ *ω*7*c*, C_17:0 cyclo_ *ω*7*c* and C_17:1_ *ω*6*c*. The dominant polar lipids were diphosphatidylglycerol, phosphatidylethanolamine, phosphatidylglycerol and an unidentified aminophospholipid. Based on the genomic physiological and phenotypic characteristics, strain Bb-Pol-6^ T^ was considered a novel species under the genus *Robbsia*, for which the name *Robbsia betulipollinis* sp. nov. was proposed. The type strain is Bb-Pol-6^ T^ (= LMG 32774^ T^ = DSM 114812^ T^).

**Supplementary Information:**

The online version contains supplementary material available at 10.1007/s00284-023-03344-7.

## Introduction

The monotypic genus *Robbsia* belongs to the class *Betaproteobacteria* [1] and the family *Burkholderiaceae* [2], which harbors highly diverse environmental bacteria including saprophytic bacteria, and a variety of pathogens that infect plants, animals and humans [2]. The phylogenetically separated species and single species in the genus, *Robbsia andropogonis*, was isolated from sorghum with strip disease and first described by Smith as *Bacterium andropogoni* [3]. The DNA G + C content ranges from 59 to 61.3 mol% [4], and is 58.92 mol% for the type strain DSM9511^T^ [5]. After initial description, *R. andropogonis* has been reclassified several times into the genus *Pseudomonas* [6], *Burkholderia* [4], *Paraburkholderia* [7], and eventually *Robbsia* proposed by Lopes-Santos et al. [8]. It is a globally distributed phytopathogen that infects a wide range of hosts including some agriculturally important crops such as maize, sorghum, common bean and tomato [9–12]. Infected leaves can develop water-soaked lesions of pale yellow or greenish white that can enlarge and collapse to involve a larger area [10]. Distinct from most phytopathogens, *R. andropogonis* strains possess a single polar sheathed flagellum [13] and are capable to produce rhizobitoxine [14]. In this study, we isolated one bacterial strain from birch (*Betula pendula*) pollen designated as Bb-Pol-6^ T^. Based on genomic and phenotypic characterization, the strain is proposed to represent a novel species within the genus *Robbsia.*

## Methods and Materials

### Strain Isolation

The pollen sample from a birch was collected from the Giessen area, Hesse state, Germany (50° 34′ 10.755″ N 8° 40′ 17.859″ E) in February 2016. The details of sample collection and strain isolation procedure were carried out according to Ambika Manirajan et al. [15]. In brief, pollen grains were collected from dissected anthers and stored in sterile tubes. The pollen sample was then suspended in 0.05% (v/v) Tween 80 and 0.18% (w/v) Na_4_P_2_O_7_, and serially diluted with 0.02% (v/v) Tween 80 and 0.085% (w/v) NaCl to 10^–5^. Dilution was plated on 1:10 AC agar medium (Sigma Aldrich), and aerobically incubated at 25 °C for one week. Strain Bb-Pol-6^ T^ was isolated by subculturing in the same medium and the purity was confirmed under light microscope. The pure culture was stored in 20% glycerol at −80 °C.

### 16S rRNA Phylogeny

Genomic DNA of strain Bb-Pol-6^ T^ was extracted from a late-log phase axenic culture according to Pitcher et al. [16]. The 16S rRNA gene was amplified by PCR using the primers 9bfm from Mühling et al. [17] and 1512uR from Weisburg et al. [18], and sequenced in both directions by LGC genomics. A consensus sequence of 1408 bp was constructed with MEGA X [19]. The retrieved 16S rRNA gene was proved to be non-chimeric using software DECIPHER 2.20.0 [20]. Next relative species and conspecific species to Bb-Pol-6^ T^ were identified by searching against the quality-controlled 16S rRNA sequence database in EzBioCloud [21] and Genbank (NCBI).

The analysis of 16S rRNA-based phylogenies was performed in ARB (version 7.0) [22] and the sequence of strain Bb-Pol-6^ T^ was aligned with related taxa and merged with the pre-aligned 16S rRNA gene database LPT_12_2021 (February 2021) [23]. Phylogenetic trees based on 16S rRNA gene sequences were reconstructed using the neighbor-joining (NJ), maximum-parsimony (MP), and maximum-likelihood (ML) methods integrated in the ARB program with 1000 replicates. The filters termini and Gap95_q0_to_q5 were applied in all tree calculations and the neighbor-joining tree was calculated with an additional Jukes-Cantor correction model and a termini filter between positions 101 and 1229 (*Escherichia coli* numbering) [24]. Maximum-parsimony and maximum-likelihood trees were calculated using the algorithms Phylip DNAPARS and RAxML 8, respectively.

### Genome Sequencing and Analysis

Extracted genomic DNA was further sequenced with the Illumina Miseq V3 (2 × 300 bp) service from LGC Genomics. The draft genome assembly was performed using SPAdes 3.15.4 [25] and open reading frame determination and gene annotation were performed using the GenDB platform [26]. Signal peptides were predicted using SignalP 6.0 [27] and CheckM was used to estimate the genome completeness and contamination [28]. The copy number of 16S rRNA gene was estimated using acn.sh tool [29]. The digital DNA-DNA hybridization between strain Bb-Pol-6^ T^ genome and the genome of the reference strain (GCA_902833845.1) was performed using formula 2 of the Genome-to-Genome Distance Calculator 3.0 with the recommended BLAST + alignment tool (https://ggdc.dsmz.de/ggdc.php) [30, 31]. Comparative genome analysis including percentage of conserved proteins (POCP), average amino acid identity (AAI), average nucleotide identity (ANI) and the calculation of genomic subsets was performed at the online platform EDGAR [32] with default settings. Secondary metabolite biosynthesis gene clusters were identified using antiSMASH 6.1.1 with strict detection strictness [33]. Resistome was predicted by CARD with default settings [34]. Plant interaction factors were detected using PIFAR-Pred on PLaBAse platform [35].

### Morphological, Physiological and Biochemical Analyses

Physiological characteristics of strain Bb-Pol-6^ T^ were determined together with the reference strain *R. andropogonis* DSM 9511^ T^. The Gram-staining was conducted as described in Gerhardt et al. [36]. Morphology of cells at late-log phase was assessed using Leica DM1000 light microscope under × 1000 magnification. Presence of flagellum was examined according to Heimbrook et al. [37] while cell motility was examined using the hanging-drop method. Catalase and oxidase activities were assessed by bubble production in 3% (v/v) H_2_O_2_ and oxidase test strips (Roth), respectively. Bacterial growth was tested on different media including R2A agar (Roth), Luria–Bertani agar (LB; Roth), trypticase soy agar (TSA; BD), nutrient agar (NA; BD), MacConkey agar (Merck) and AC 1:10 agar (Sigma). The optimal growth conditions were assessed by incubation in AC 1:10 broth at a range of temperatures (4, 15, 20, 28, 33, 37, 45 °C), pH (2.0–11.0, at intervals of 0.5) and salinity (0–5.0% w/v NaCl, at intervals of 0.5%). The broth pH-value was adjusted by 0.2 M Na_2_HPO_4_/0.4 M citrate for pH 2.0–8.0, and 0.2 M glycine/0.4 M NaOH for pH 8.5–11.0 with slight modification as described by Lin et al*.* [38]. Before the growth success was controlled, strain Bb-Pol-6^ T^ and *R. andropogonis* DSM 9511^ T^ were grown for five and three days respectively, except for inoculants at 4 °C, which were grown for two weeks. Strain Bb-Pol-6^ T^ was incubated on AC 1:10 agar medium anaerobically using Anaerocult A system (Merck) to examine its oxygen requirement. Hypersensitive response was examined on tobacco (*Nicotiana tabacum* cv. Xanthi) and birch (*Betula pendula*) according to Klement and Goodman [39]. Rhizobitoxine production bioassay was performed based on Ruan and Peters [40]. Biochemical characteristics including enzyme activities and assimilation of carbon sources were determined with API ZYM, API 20 NE and API 50 CH strips (bioMérieux) according to the manufacturer’s instructions. As strain Bb-Pol-6^ T^ is a slow-grower, the incubation time of assimilation tests on API 20 NE and API 50 CH strips for strain Bb-Pol-6^ T^ was extended to five days for a clear result. The assimilation of acetate, succinate, malonate and propionate that are not involved in API system was also tested on API AUX medium.

### Chemotaxonomic Characteristics

Cellular fatty acid, respiratory quinones and polar lipid composition of strain Bb-Pol-6^ T^ and the reference strain were analyzed using corresponding DSMZ services. Late-log phase bacterial culture in AC 1:10 medium was harvested and freeze dried. Cellular fatty acids were converted into fatty acid methyl esters (FAMEs) using a modified method of Miller [41] and Kuykendall et al. [42], separated by gas chromatography and detected using Sherlock Microbial Identification System (MIDI, Microbial ID). Summed features are resolved and identities of fatty acids are confirmed by a GC–MS-based analysis. Respiratory quinones were extracted using hexane [43, 44], purified by a silica-based solid phase extraction, and analyzed by HPLC. Polar lipids were extracted based on Bligh and Dyer [45], separated by two-dimensional silica gel thin layer chromatography, and detected according to Tindall et al. [46].

### Repositories

The GenBank/EMBL/DDBJ accession number for the 16S rRNA gene sequence of strain Bb-Pol-6^ T^ is KX450422. The whole-genome shotgun BioProject number is PRJNA875284 with the GenBank assembly accession number JAPMXC000000000. The strain has been deposited in the DSMZ under the number DSM 114812, and the BCCM/LMG under the number LMG 32774.

## Results and Discussion

### Isolation

From the same birch pollen sample where Bb-Pol-6^ T^ was isolated, one gram of sample was found to contain 4.1 ± 3.1 × 10^5^ colony forming units (CFU) [15]. Culture-dependent method revealed 15 bacterial species from four phyla: *Proteobacteria*, *Actinobacteria*, *Firmicutes* and *Bacteroidetes*. Culture-independent method revealed 441 operational taxonomic units (OTU), and the most dominant phyla were *Proteobacteria*, *Actinobacteria* and *Acidobacteria* [15]*.* Compared to insect-pollinated pollen species (autumn crocus and rape), birch pollen possesses significantly higher species richness and different communities [15]. Furthermore, it was shown that bacteria and pollen from wind pollinated plants like birch could have a higher allergic potential [47]. This possible higher allergic potential is caused by a high lipopolysaccharide concentration (LPS) concentration of the bacteria on the pollen. Interestingly, strain Bol-Pol-6^ T^ has one of the highest measured LPS concentrations in this study with an LPS concentration of 76.6 ng l^−1^ [47] and five next relative uncultured bacteria (pairwise similarity values 98.72–99.70%) found in floor dust (FM872738) [48] and on human skin (HM270658, HM270467, JF167742, JF152894) [49] are also associated to allergic reaction in these studies.

### 16S rRNA Phylogeny

According to the highest pairwise gene similarity results in EzBioCloud (Ez) and ARB (Table S1), *R. andropogonis* DSM 9511^ T^ (ARB 95.9, Ez 96.23), *Chitinasiproducens palmae* JS23^T^ (ARB 96, Ez 95.94), *Pararobbsia silviterrae* DHC34^T^ (ARB 95.7, Ez 95.58) *Paraburkholderia elongata* 5N^T^ (ARB 95.6, Ez 95.51) were identified as the next-relative species with valid names to strain Bb-Pol-6^ T^. As the similarity is less than 98.7%, strain Bb-Pol-6^ T^ can be considered a potential novel species [50]. Similarities with five conspecific species range from 98.72% to 99.70%. The ML tree (Fig. S1) showed that strain Bb-Pol-6^ T^ and its conspecific species were clustered with *R. andropogonis* DSM 9511^ T^, forming a stable monophyletic group, which was consistently revealed in the NJ and MP trees (Figs. S2 and S3). This indicates the potential phylogenetic position of strain Bb-Pol-6^ T^ under the genus *Robbsia*.

### Genome Sequencing and Analysis

**Fig. 1:**
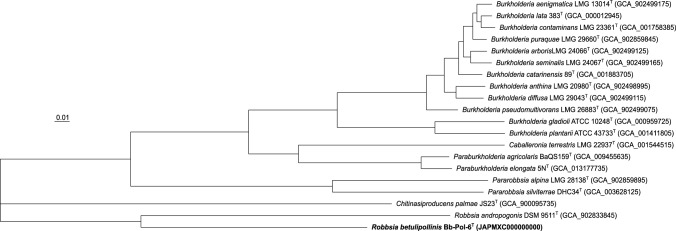
Maximum-likelihood tree built out of a core of 1307 genes of the genomes showing the phylogenetic position of strain Bb-Pol-6^ T^ in relation to 19 most closely related species. Bar, 0.01 substitutions per nucleotide position

The draft genome assembly of strain Bb-Pol-6^ T^ consisted of 46 contigs with a total size of 5.04 Mbp and the longest contig was 2.68 Mbp which was the N50 length. This genome contained 4401 predicted coding sequences among which 509 sequences were predicted to encode signal peptides, 57 tRNA genes and three rRNA genes, and had a G + C content of 65.31 mol%. Completeness and contamination of the genome was 98.44 and 0.05%, respectively. Although four copies of 16S rRNA gene were predicted only one 16S rRNA gene sequence from the draft genome was partially recovered with a length of 470 bp. *R. andropogonis* DSM 9511^ T^ has the closest AAI and ANI values to strain Bb-Pol-6^ T^ (72.42% and 72.53%) compared to the other next-relatives (Fig. S4). The dDDH and POCP values of strain Bb-Pol-6^ T^ to *R. andropogonis* DSM 9511^ T^ were 22.7 and 65.85%, respectively. These values were below the generally accepted species boundary values (AAI and ANI: 95%; dDDH: 70%) [51, 52], and above the proposed genus boundary value (POCP: 50%) [53], thus rendering strain Bb-Pol-6^ T^ a promising novel species. The ML tree calculated from the genome of strain Bb-Pol-6^ T^ and 19 closest-related species using 1307 core genes again revealed the monophyletic group formed by strain Bb-Pol-6^ T^ and *R. andropogonis* DSM 9511^ T^, confirming its phylogenetic position within the genus *Robbsia* (Fig. 1). Comparative genome analysis of strain Bb-Pol-6^ T^ and *R. andropogonis* DSM 9511^ T^ showed 7221 pan genes, 2720 core genes and 1186 strain Bb-Pol-6^ T^ singleton genes. Identified secondary metabolite gene clusters by antiSMASH included non-ribosomal peptide synthetase (NRPS), redox-cofactor, phosphonate and terpene (Fig. S5). Two antibiotic resistance ontology terms with antibiotic efflux mechanism, qacG and adeF, were predicted using protein homolog model in CARD. The most abundant genes annotated by PIFAR-Pred were toxin-encoding genes (35%) (Fig. S6). Among these toxin-encoding genes, the majority encode syringomycin, a necrosis-inducing phytotoxin [54], and toxoflavin, a virulence factor of phytopathogens such as *Burkholderia glumae* in crop diseases [55, 56], indicating the phytopathogenic potential of strain Bb-Pol-6^ T^.

### Morphological, Physiological and Biochemical Analyses

Cells of strain Bb-Pol-6^ T^ were rod-shaped (0.4–0.7 µm in width and 0.9–1.6 µm in length) with no flagellum, Gram-stain-negative, facultative anaerobic, non-spore-forming and non-motile. Colonies of strain Bb-Pol-6^ T^ were round, convex, viscous, creamy in color and has entire margin after grown on AC 1:10 agar for five days. The strain grew optimally on AC 1:10 agar and growth was also observed on R2A agar, but not on LB, TSA, NA and MacConkey agar. Strain Bb-Pol-6^ T^ was catalase-positive and oxidase-negative. The temperature, salinity and pH value ranges for growth were 4–28 °C (optimum at 28 °C), 0–1% NaCl (optimum without NaCl) and 5–7.5 pH (optimum at 6–7). Unlike *R. andropogonis*, strain Bb-Pol-6^ T^ cannot induce hypersensitive response on *Nicotiana tabacum* cv. Xanthi and *Betula pendula* (Fig. S7) and does not produce rhizobitoxine. The differential physiological and biochemical characteristics of strain Bb-Pol-6^ T^ and its reference strain were summarized in Table 1.

**Table 1:** Differential characteristics between strain Bb-Pol-6^ T^ and its reference type strain

Characteristics	1	2
Motility	–	+
Hypersensitive response	–	+
Flagellum	–	+
Rhizobitoxine production	–	+
Growth at:		
Temperature range (°C) (optimum)	4–28 (28)	15–33 (28)
pH range (optimum)	5–7.5 (6–7)	4–10 (7–8)
NaCl range (optimum)	0–1 (0)	0–4 (0–1)
Enzyme activity		
α-Glucosidase	+	–
Alkaline phosphatase	–	+
Assimilation of:		
D-Adonitol, D-lyxose, D-sorbitol, inositol, D-arabinose, malonate, acetate	–	+
DNA G + C content (mol%)	65.31	58.92

Strains: 1, Bb-Pol-6^ T^; 2, *Robbsia andropogonis* DSM 9511^ T^. All data were obtained from this study (+ , positive; −, negative), except the DNA G + C content of *R. andropogonis* DSM 9511^ T^ [5]. All strains were Gram-stain-negative, oxidase-negative and catalase positive

### Chemotaxonomic Characteristics

On AC 1:10 medium, ubiquinone 8 was the major respiratory quinone of both strain Bb-Pol-6^ T^ (96.5%) and *R. andropogonis* DSM 9511^ T^ (97.7%). The major cellular fatty acids of both strains were C_16:0_, C_19:0 cyclo_ *ω*7*c*, C_17:0 cyclo_ *ω*7*c* and C_17:1_ *ω*6*c* (Table S2). Compared to *R. andropogonis* DSM 9511^ T^, strain Bb-Pol-6^ T^ contained a higher amount of C_12:0_ (2.8%), C_18:1_ *ω*5*c* (2.9%) and C_16:1_ *ω*7*c* (8.8%), and a lower amount of C_14:0_ (0.4%), C_17:0_ (0.7%) and C_18:1_ *ω*7*c* (0.3%). Strain Bb-Pol-6^ T^ contained trace amount of C_10:0_, C_12:0_ 3-OH and C_18:1_ *ω*7*c* 11-methyl, which were not detected in *R. andropogonis* DSM 9511^ T^, while C_15:0_ 3-OH detected in *R. andropogonis* DSM 9511^ T^ was not present in strain Bb-Pol-6^ T^. The major polar lipids in strain Bb-Pol-6^ T^ were diphosphatidylglycerol, phosphatidylethanolamine, phosphatidylglycerol, one unidentified aminolipid, one unidentified aminophospholipid, two unidentified phospholipids and six unidentified lipids (Fig. S8). Glycolipid was identified in *R. andropogonis* DSM 9511^ T^ but not in strain Bb-Pol-6^ T^.

## Conclusions

In summary, the phylogenetic analysis revealed that strain Bb-Pol-6^ T^ belongs to the genus *Robbsia*. Further phenotypic and biochemical characterization distinguished strain Bb-Pol-6^ T^ from its next-relative reference strain *R. andropogonis* DSM 9511^ T^ suggesting it represents a novel species, for which the name *Robbsia betulipollinis* sp. nov. is proposed.

### Description of *Robbsia betulipollinis* sp. nov

*Robbsia betulipollinis* (be.tu.li.pol′li.nis L. n. *betula* birch; L. n. *pollen* pollen; N.L. gen. n. *betulipollinis* of pollen from birch).

Cells are Gram-negative, facultative anaerobic, non-spore-forming, non-motile, rod-shaped (0.4–0.7 µm wide × 0.9–1.6 µm long) and do not have flagellum. Colonies were round, convex, viscous, creamy in color and has entire margin after five days of incubation on AC 1:10 agar. Grows between 4 and 28 °C (optimum at 28 °C), 0–1% NaCl (optimum without NaCl) and 5–7.5 pH (optimum at 6–7). Positive for catalase, esterase (C4), esterase (C8), acid phosphatase, β-galactosidase, leucine arylamidase, valine arylamidase, naphthol-AS-BI-phosphohydrolase, α-Glucosidase, D-galactose, D-mannitol, D-mannose, D-ribose, L-arabinose, D-arabitol, D-glucose, D-fructose and succinate. Negative for oxidase, nitrate reduction, glucose fermentation, arginine dihydrolase, gelatin hydrolysis, esculin hydrolysis, indole production, cystine arylamidase, trypsin, α-chymotrypsin, α-galactosidase, N-acetyl-β-glucosaminidase, lipase (C 14), β-glucuronidase, β-glucosidase, α-mannosidase, α-fucosidase, urease, alkaline phosphatase, adipic acid, amygdalin, arbutin, capric acid, cellobiose, D-fucose, D-gentiobiose, lactose, maltose, melezitose, melibiose, raffinose, sucrose, D-tagatose, turanose, dulcitol, D-xylose, glycerol, inulin, L-arabitol, L-fucose, L-rhamnose, L-Xylose, malic acid, methyl α-D-glucopyranoside, methyl α-D-mannopyranoside, N-acetyl-D-glucosamine, phenylacetic acid, potassium 2-ketogluconate, potassium 5-ketogluconate, potassium gluconate, salicin, starch, xylitol, esculin ferric citrate, trehalose, glycogen, erythritol, methyl-ß-D-xylopyranoside, L-sorbose, trisodium citrate, propionate, acetate, D-adonitol, D-lyxose, D-sorbitol, inositol, D-arabinose, malonate, hypersensitive response and rhizobitoxine production. The major respiratory quinone is ubiquinone 8. The major cellular fatty acids are C_16:0_, C_19:0 cyclo_ *ω*7*c*, C_17:0 cyclo_ *ω*7*c* and C_17:1_ *ω*6*c*. The major polar lipids are diphosphatidylglycerol, phosphatidylethanolamine, phosphatidylglycerol, one unidentified aminolipid, one unidentified aminophospholipid, two unidentified phospholipids and six unidentified lipids.

The type strain, Bb-Pol-6^ T^ (= LMG 32774^ T^ = DSM 114812^ T^), was isolated from pollen of birch in Giessen, Hesse, Germany (50° 34′ 10.755″ N 8° 40′ 17.859″ E). The genome of the type strain has a size of 5.04 Mbp with a G + C content of 65.31 mol%.

## Supplementary Information

Below is the link to the electronic supplementary material.

Supplementary file1 (PDF 1384 KB)

## Data Availability

The data used and/or analysed during the current study are available from the corresponding author on reasonable request.
