# Supplementary material for: Robbsia betulipollinis sp. nov., Isolated from Pollen of Birch (Betula pendula)
Source: Curr Microbiol. 2023 Jun 6;80(7):234. doi: 10.1007/s00284-023-03344-7 (PMC10244264; doi:10.1007/s00284-023-03344-7)
Supplement: Supplementary file 1 — Supplementary file1 (PDF 1384 KB) [file 284_2023_3344_MOESM1_ESM.pdf]

Supplement Material

Current Microbiology

Robbsia betulipollinis sp. nov., isolated from pollen of birch (*Betula pendula*)

Haoran Shi[1], Binoy Ambika Manirajan[1,2], Stefan Ratering[1,*], Rita Geissler-Plaum[1] and Sylvia Schnell[1]

[1]Institute of Applied Microbiology, Research Center for BioSystems, Land Use, and Nutrition (IFZ), Justus-Liebig University Giessen, 35392 Giessen, Germany

[2]School of Biosciences, Mahatma Gandhi University, Kerala, India.

Table S1. The 16S rRNA pairwise gene similarity values for the strain Bb-Pol-6^{T} with closest related 16S rRNA gene bacteria sequences of bacteria with recognized prokaryotic names retrieved from GenBank database and EzBioCloud database. Similarity analysis was performed using ARB version 7 and EzBioCloud.

**Next relative strain, (accession number)**	ARB (Bb-Pol-6^{T})	EzBioCloud (Bb-Pol-6^{T})
***Chitinasiproducens palmae*, JS23^{T}, (KT337490)**	96.0	95.94
***Robbsia andropogonis*, DSM 9511^{T}, (JX986957)**	95.9	96.23
***Pararobbsia alpina*, PO-04-17-38^{T}, (JF763852)**	95.9	95.48
***Pararobbsia silviterrae*, DHC34^{T,} (KP938219)**	95.7	95.58
***Paraburkholderia elongata*, 5N^{T}, (MN723157)**	95.6	95.51
***Burkholderia gladioli*, CIP 105410^{T}, (EU024168)**	95.4	95.16
***Burkholderia plantarii*, LMG 9035^{T}, (U96933)**	95.4	95.44
***Paraburkholderia agricolaris*, BaQS159^{T}, (NZ_QPER01000001.1)**	95.3	95.23
***Burkholderia anthina*, LMG 20980^{T,} (HQ849074)**	95.3	95.09
***Burkholderia pseudomultivorans*, LMG 26883^{T}, (HE962386)**	95.2	95.29
***Burkholderia puraquae*, CAMPA 1040^{T}, (KX278717)**	95.2	95.30
***Burkholderia diffusa*, R-15930^{T}, (AM747629)**	95.2	95.30
***Burkholderia catarinensis*, 89^{T}, (KR013050)**	95.2	95.30
***Caballeronia terrestris*, LMG 22937^{T}, (HE981726)**	95.1	95.09
***Burkholderia seminalis*, R-24196^{T}, (AM747631)**	95.1	95.22
***Burkholderia contaminans*, LMG 23361^{T}, (JX986975)**	95.1	95.23
***Burkholderia lata*, 383^{T}, (CP000150)**	95.1	95.16
***Burkholderia arboris*, R-24201^{T}, (AM747630)**	95.1	95.22

Table S2. Cellular fatty acid composition of strain Bb-Pol-6T and its next relative Strains: 1, Bb-Pol-6T; 2, Robbsia andropogonis DSM 9511T. All data were obtained from this study. Strains were cultured in AC 1:10 liquid medium at 28 °C with shaking. Values are percentages of the total fatty acids.

**Fatty acids**	**1**	**2**
$C_{12:0}$	2.8	0.1
$C_{14:0}$	0.4	3.1
$C_{16:0}$	12.5	16.4
$C_{17:0\ cyclo}$ $\omega 7c$ and $C_{17:1}$ $\omega 6c$	18.4	21.7
$C_{17:0}$	0.7	3.0
$C_{16:1}$ $\omega 7c$ 2-OH	5.2	3.1
$C_{16:0}$ 2-OH	2.4	3.3
$C_{16:0}$ 3-OH	3.8	3.8
$C_{18:1}$ $\omega 5c$	2.9	0.9
$C_{18:0}$	0.6	1.3
$C_{19:0\ cyclo}$ $\omega 7c$	35.0	27.3
$C_{14:0}$ 3-OH	2.5	3.5
$C_{16:1}$ $\omega 7c$	8.8	4.3
$C_{18:1}$ $\omega 7c$	0.3	4.4

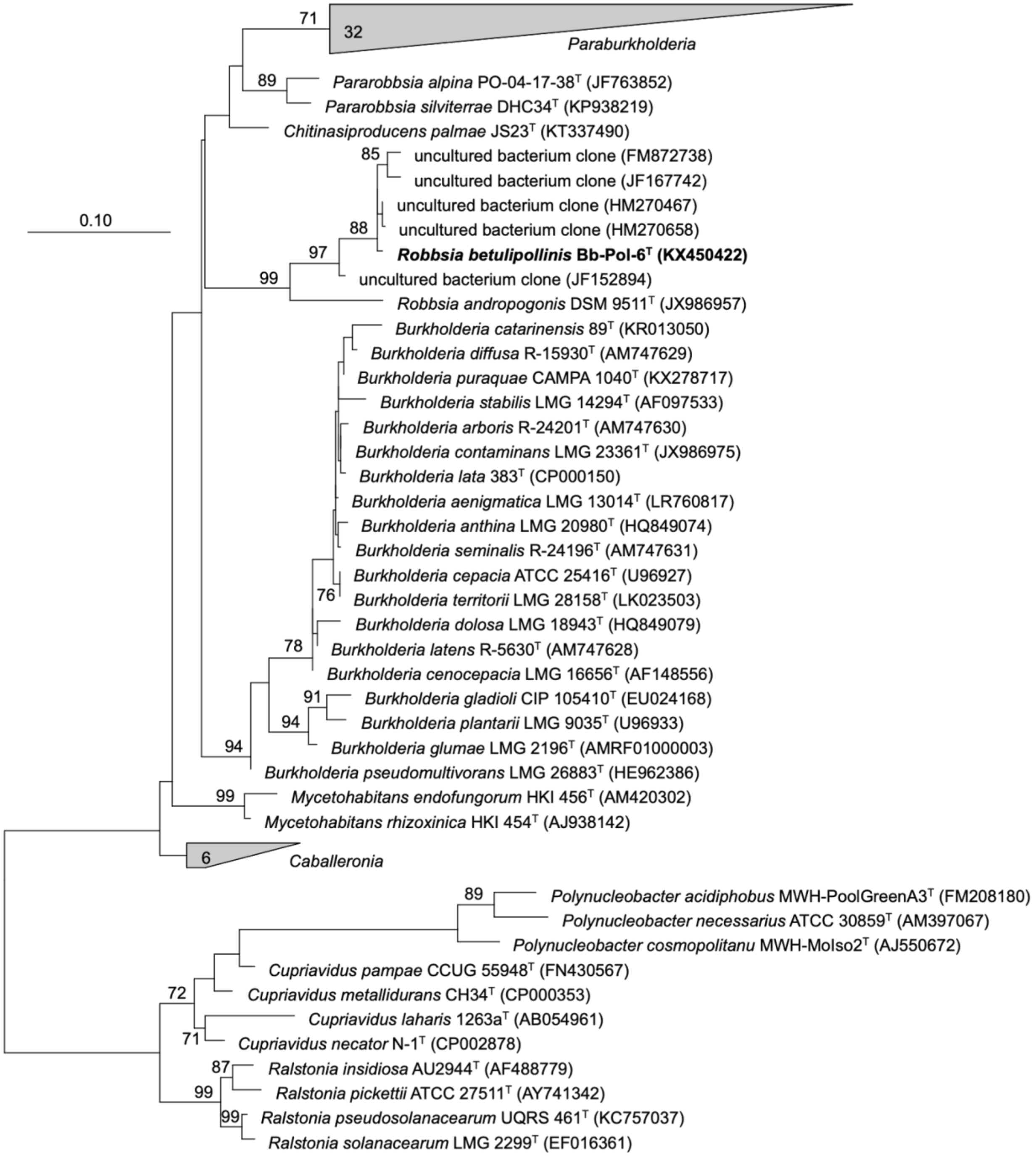

Fig. S1. A maximum-likelihood phylogenetic tree based on comparative analysis of 16S rRNA gene sequences, showing the phylogenetic position of the strain Bb-Pol-6^T among related type strains and conspecific species. *Polynucleobacter*, *Cupriavidus* and *Ralstonia* were used to root the tree. Bootstrap value (>70%) based on 1000 replicates are shown at the branch points. Bar: 0.10 substitutions per nucleotide position.

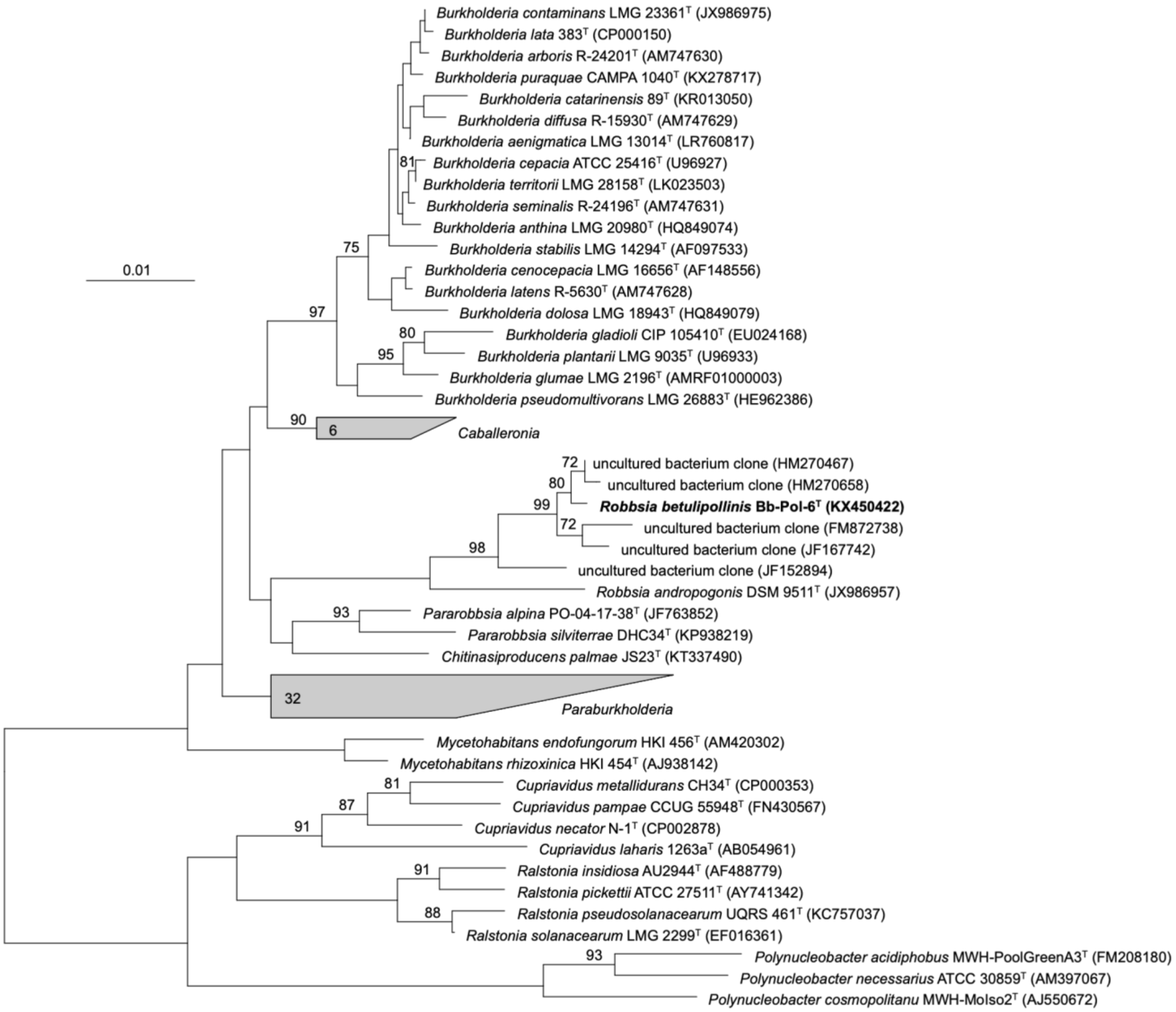

Fig. S2. A neighbor-joining phylogenetic tree based on comparative analysis of 16S rRNA gene sequences, showing the phylogenetic position of the strain Bb-Pol-6^T among related type strains and conspecific species. *Polynucleobacter*, *Cupriavidus* and *Ralstonia* were used to root the tree. Bootstrap value (>70%) based on 1000 replicates are shown at the branch points. Bar: 0.01 substitutions per nucleotide position.

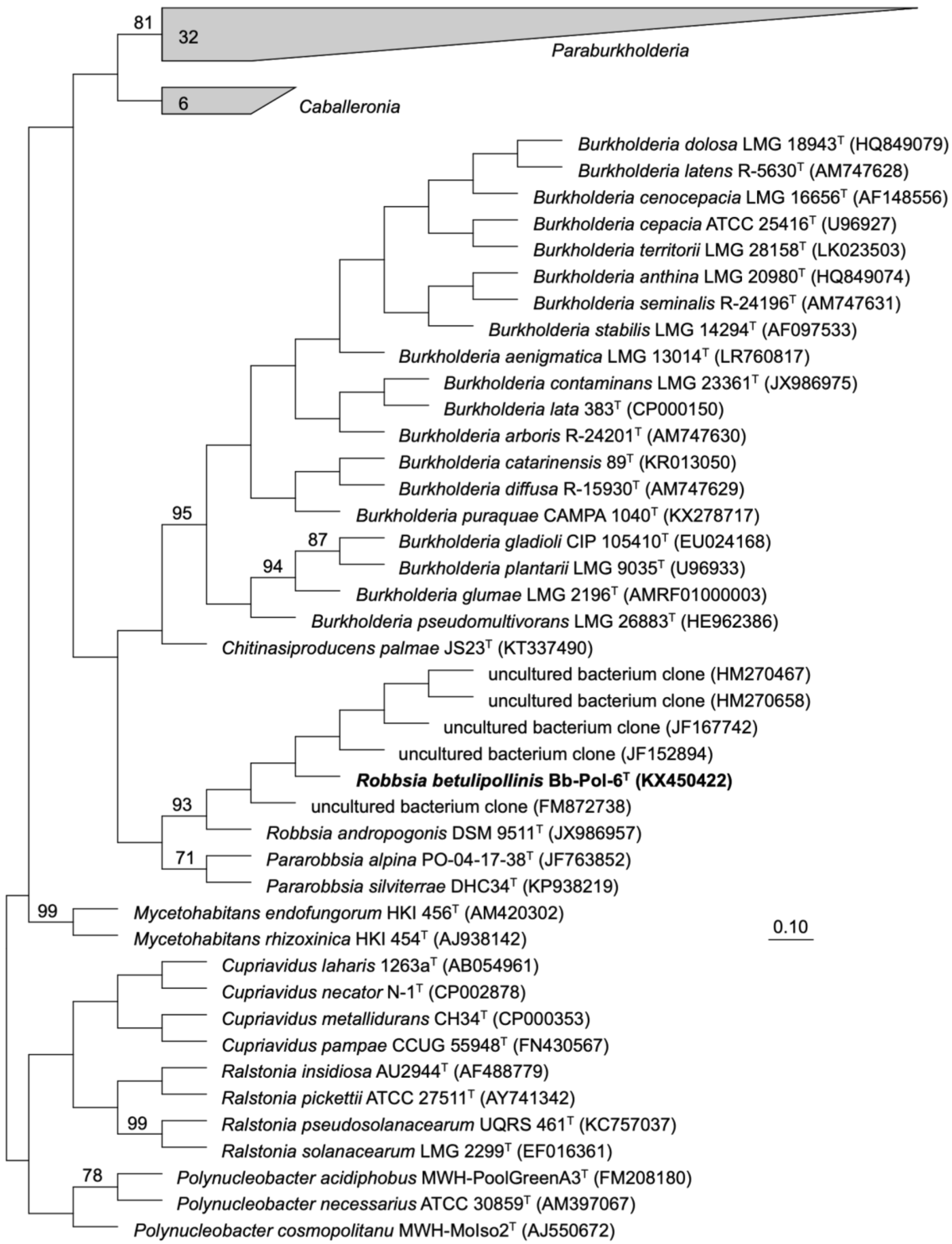

Fig. S3. A maximum-parsimony phylogenetic tree based on comparative analysis of 16S rRNA gene sequences, showing the phylogenetic position of the strain Bb-Pol-6^{T} among related type strains and conspecific species. *Polynucleobacter*, *Cupriavidus* and *Ralstonia* were used to root the tree. Bootstrap value (>70%) based on 1000 replicates are shown at the branch points. Bar: 0.10 substitutions per nucleotide position.

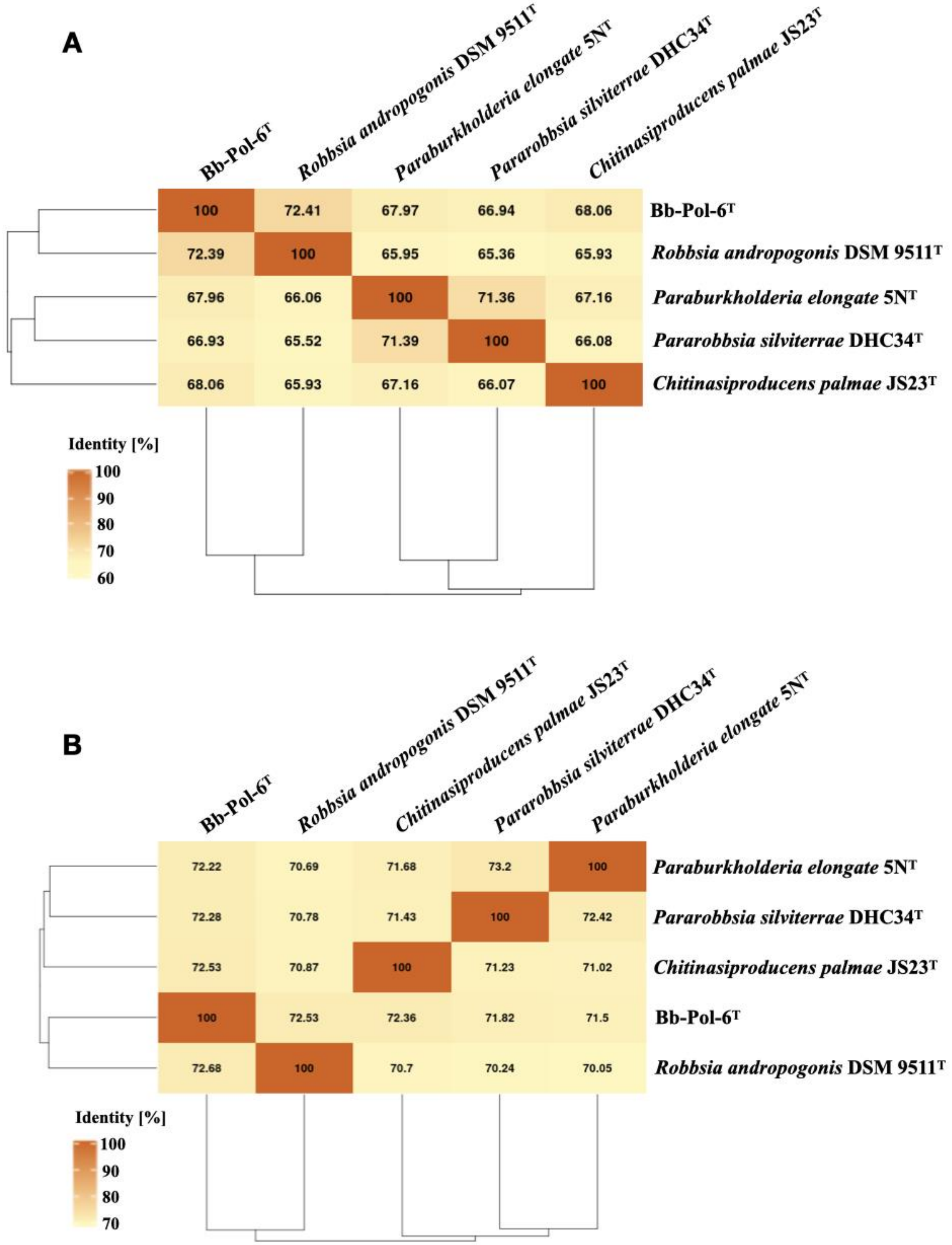

Fig. S4. Average amino acid identity (A) and average nucleotide identity (B) mean values matrix heatmaps between the strain Bb-Pol-6^{T} and the next-relative strains *Robbsia andropogonis* DSM 9511^{T}, *Chitinasiproducens palmae* JS23^{T}, *Pararobbsia silviterrae* DHC34^{T} and *Paraburkholderia elongate* 5N^{T}.

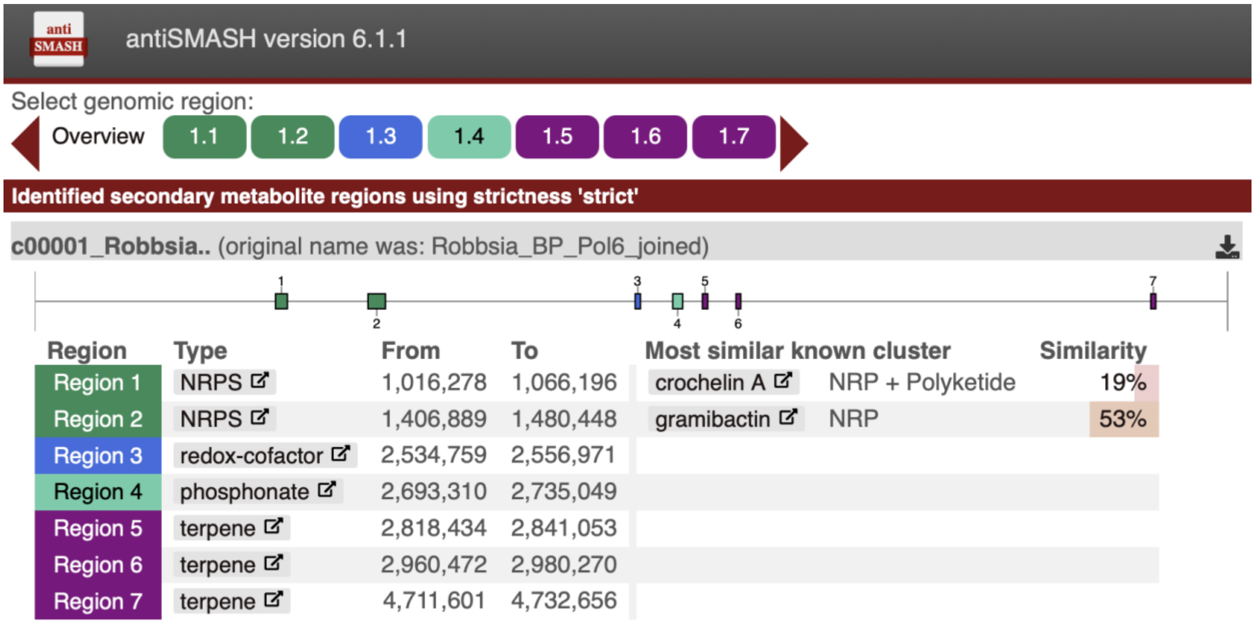

Region	Type	From	To	Most similar known cluster		Similarity
Region 1	NRPS	1,016,278	1,066,196	crochelin A	NRP + Polyketide	19%
Region 2	NRPS	1,406,889	1,480,448	gramibactin	NRP	53%
Region 3	redox-cofactor	2,534,759	2,556,971			
Region 4	phosphonate	2,693,310	2,735,049			
Region 5	terpene	2,818,434	2,841,053			
Region 6	terpene	2,960,472	2,980,270			
Region 7	terpene	4,711,601	4,732,656			

Fig. S5. Identified secondary metabolite gene clusters in the draft genome of strain Bb-Pol-6^T using antiSMASH. Four gene clusters were identified including non-ribosomal peptide synthetase (NRPS), redox-cofactor, phosphonate and terpene.

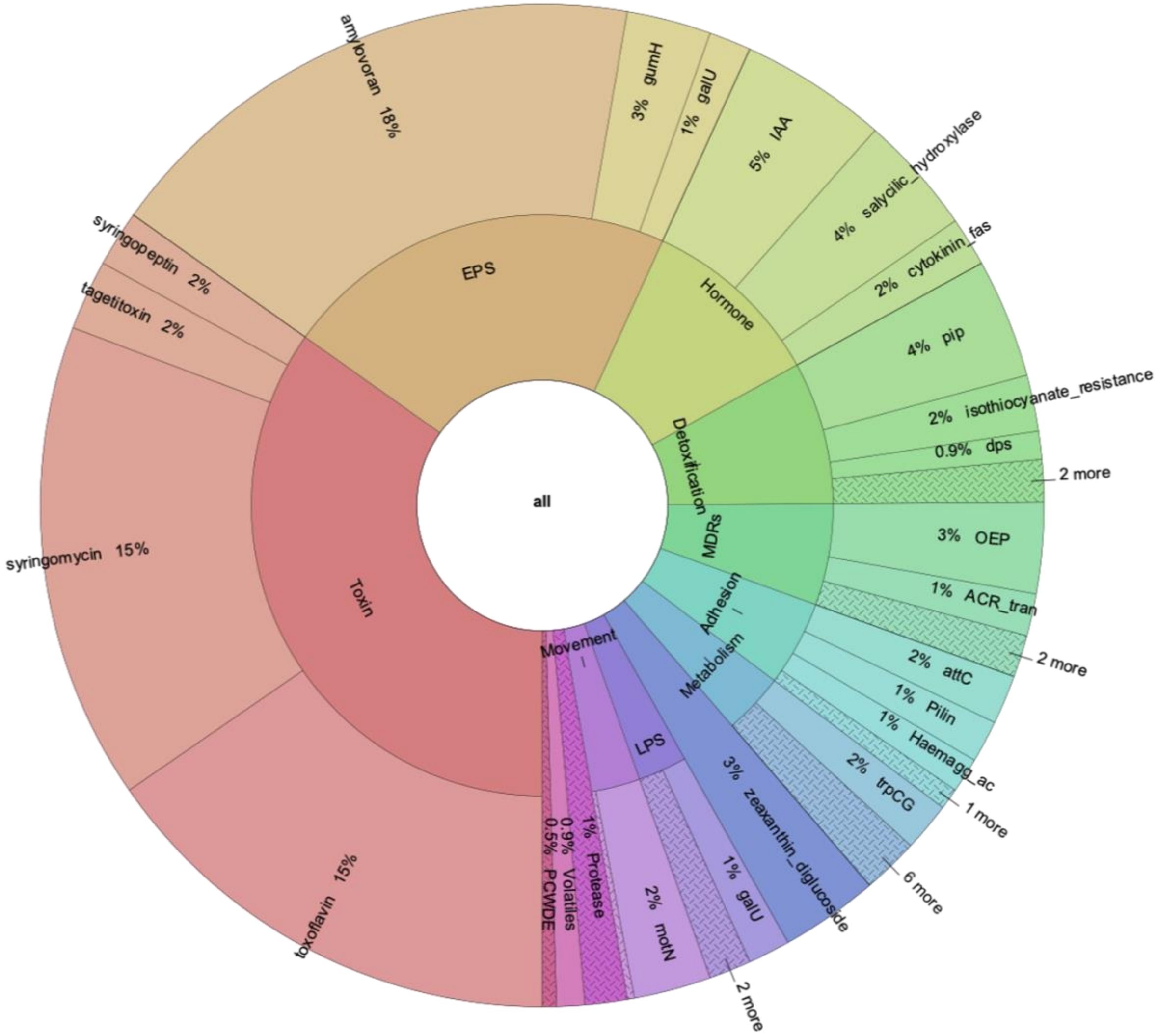

Fig. S6. Genes annotated by PIFAR-Pred from the draft genome of strain Bb-Pol-6^{T}. The major genes are toxin-encoding genes including syringomycin (15%) and toxoflavin (15%). EPS, exopolysaccharide; IAA, indole-3-acetic acid; MDR, multidrug resistance; OEP, outer membrane efflux proteins; LPS, lipopolysaccharide; PCWDE, Plant cell wall-degrading enzyme.

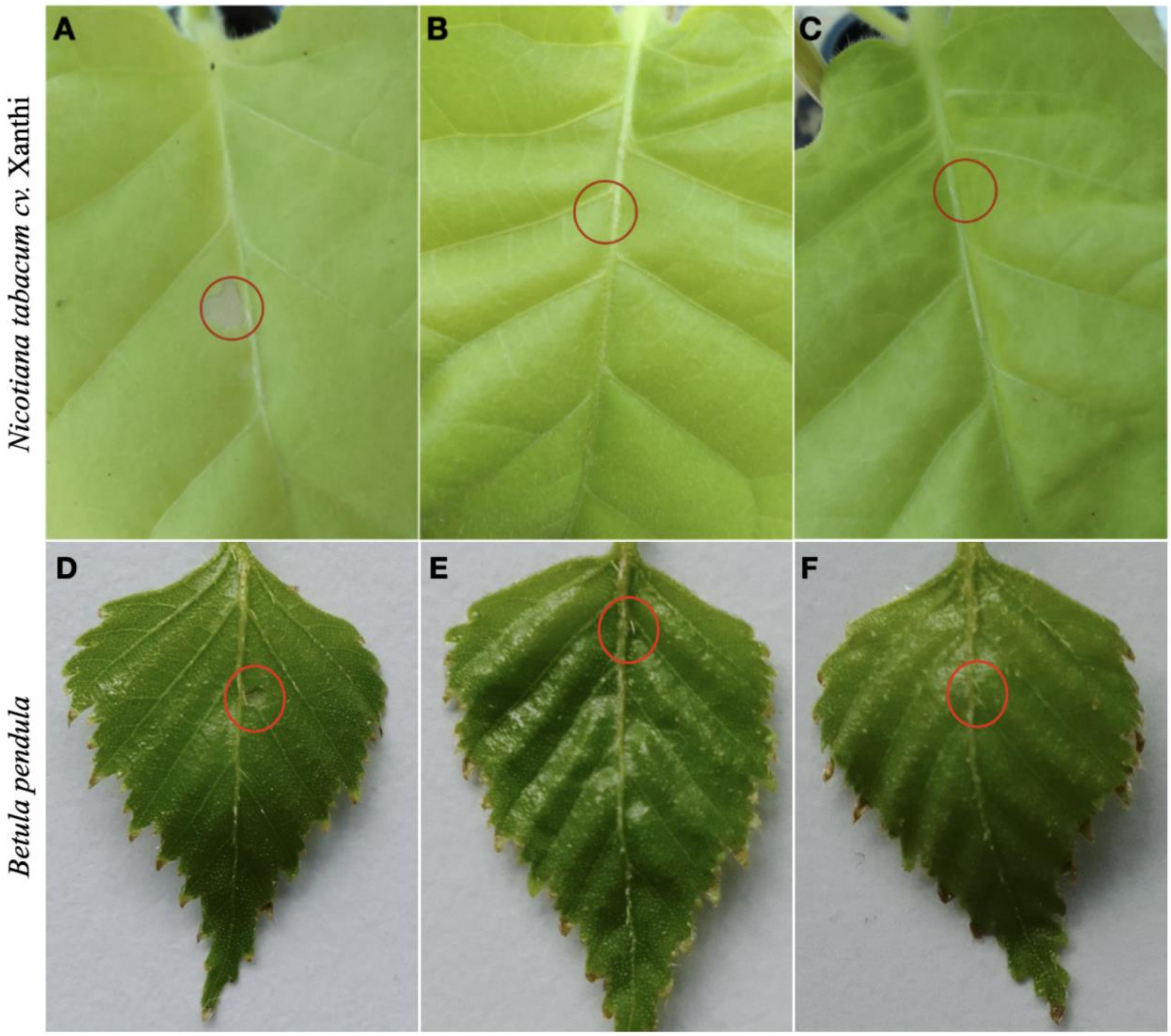

Fig. S7. Hypersensitive response assay of *Robbsia andropogonis* DSM 9511^{T} (A and D), *Robbsia* Bb-Pol-6^{T} (B and E) and phosphate buffer control (C and F) on tobacco (*Nicotiana tabacum* cv. Xanthi) and birch (*Betula pendula*) leaves. Red circle indicates the injection spot.

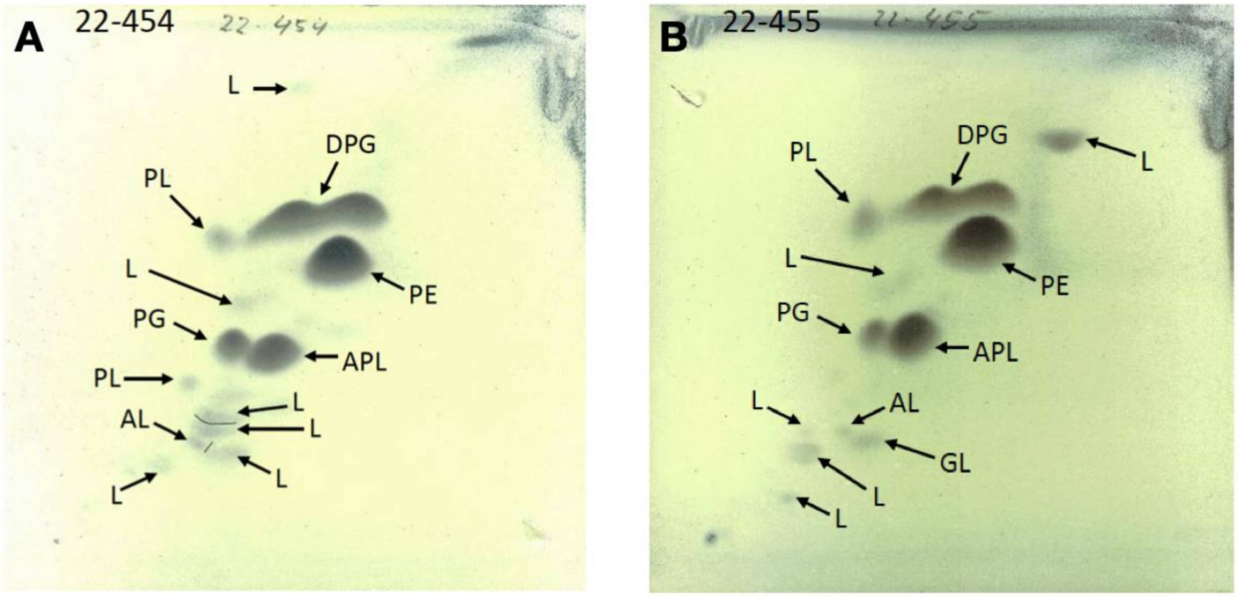

Fig. S8. Polar lipids profile of strain Bb-Pol-6^T (A) and *Robbsia andropogonis* DSM 9511^T (B) separated by two-dimensional silica gel thin layer chromatography. DPG, diphosphatidylglycerol; PE, phosphatidylethanolamine; PG, phosphatidylglycerol; AL, aminolipid; APL, aminophospholipid; PL, phospholipid; L, lipid; GL, Glycolipid.
